# Global, regional and national burden of rheumatoid arthritis from 1990 to 2021, with projections of incidence to 2050: a systematic and comprehensive analysis of the Global Burden of Disease study 2021

**DOI:** 10.1186/s40364-025-00760-8

**Published:** 2025-03-24

**Authors:** Yingnan Ma, Haiyan Chen, Wenhua Lv, Siyu Wei, Yuping Zou, Ruilin Li, Jiacheng Wang, Wei She, Linna Yuan, Junxian Tao, Xuying Guo, Shuo Bi, Hongsheng Tian, Ye Ma, Hongmei Sun, Chen Sun, Jing Xu, Yu Dong, Jingxuan Kang, Hongchao Lv, Mingming Zhang, Yongshuai Jiang

**Affiliations:** 1 College of Bioinformatics Science and Technology, Harbin Medical University, 194 Xuefu Road, Nangang District Heilongjiang Province, Harbin, China; 2 RABC: Rheumatoid Arthritis Bioinformatics Center, Harbin, China; 3 Faculty of Information Engineering and Automation, Kunming University of Science and Technology, Kunming, China

**Keywords:** Rheumatoid arthritis, Global burden of disease, Autoimmune disease, Public health

## Abstract

**Background:**

To provide insights into rheumatoid arthritis (RA) epidemiological trends, including prevalence, incidence, disability-adjusted life years (DALYs), corresponding average annual percentage change (AAPC), gender disparities, regional variations, age-specific rates, socio-economic correlations, risk factors, and future projections.

**Methods:**

Data were extracted from the Global Burden of Disease Study (GBD) 2021. AAPC was calculated by joinpoint regression and two-sample Mendelian randomization (MR) analysis was performed to verify the causal relationship between the smoking factor and RA. The future incidence trend was predicted by the Bayesian age-period-cohort (BAPC) model.

**Results:**

Global age-standardized prevalence rate (ASPR) and age-standardized incidence rate (ASIR) increased significantly while age-standardized DALYs rate (ASDR) decreased from 1990 to 2021. Regional variations were pronounced, with Andean Latin America reporting the highest burden. Females consistently exhibited higher age-standardized rate (ASR) across all metrics. Age-specific prevalence, incidence, and DALYs rates peaked at different age groups, highlighting complex demographic dynamics. Socio-demographic index (SDI) analysis demonstrated a positive correlation between RA burden and socio-economic development. The two-sample MR analysis confirmed a causal effect between smoking and RA. From 2022 to 2050, the ASIR will increase moderately.

**Conclusions:**

The study underscores the escalating burden of RA globally, emphasizing the need for healthcare providers to be aware of the effects of aging populations and other societal factors on the risk of developing RA, and to develop targeted interventions, including smoking cessation programs, age- and gender-appropriate healthcare, and early diagnosis strategies.

**Supplementary Information:**

The online version contains supplementary material available at 10.1186/s40364-025-00760-8.

## Background

Rheumatoid arthritis (RA) is a typical autoimmune disease that mainly affects multiple joints [1, 2] and is characterized by symmetrical, invasive inflammatory lesions in multiple surrounding joints. The global incidence of RA is approximately 0.24% to 1% [3]. With the development of the disease, the joint tissues of patients in later periods are severely damaged, deformed, and even disabled, and systemic complications such as infection, lymphoma, osteoporosis, and cardiovascular disease will also be caused [4]. RA has become a global public health problem, therefore, analysis of the global disease burden for RA is crucial.

The Global Burden of Disease Study (GBD) 2021 is a valuable tool for investigating the burden of disease. Nowadays, there are some studies based on GBD 2021 or earlier versions to report on the burden of RA. A previous study reported the global burden of autoimmune diseases and cross-country inequalities, but it did not provide a systematic analysis for RA [5]. One study reported the global burden of RA from 1990 to 2017, but it included fewer countries and did not provide a detailed analysis of risk factors [6]. Another report using the GBD 2019 analyzed the global burden of RA as well as risk factors, but it didn’t predict future incidence trends [7]. A study published in 2023 used GBD 2021 to analyze the burden of RA from 1990 to 2020, yet in 2021 the number of prevalence cases increased again by about 320,000, which was not reflected in the study [8]. The GBD is based on model estimates, and its data change with each update. The data in the old version of the GBD are outdated, and the current analysis of the global burden of RA using the latest version of the GBD is not comprehensive, which might limit the establishment of effective measures at global, regional, and national levels. With the increase of public health data on RA worldwide and the update of the GBD database, a more systematic and comprehensive analysis is urgently needed to develop strategies on the global, national, and regional scale.

Hence, this study aims to provide the most comprehensive, detailed, and up-to-date report on the global burden of RA based on GBD data at the global, regional, and national levels from 1990 to 2021. Here, we provided a detailed comparative analysis for RA about prevalence, incidence, disability-adjusted life years (DALYs), and the influence of age, gender, and socio-demographic index (SDI) at global, national, and regional levels. In addition, we also discussed the contribution of risk factors to RA DALYs and predicted the future global incidence trend of RA.

## Methods

### Study population and data collection

All data used in the study were extracted from GBD 2021, conducted by the Institute of Health Metrics and Evaluation (IHME), which assessed the burden of 371 diseases and injuries and 88 risk factors for 204 countries and territories, 28 GBD super regions and 54 GBD regions during the period 1990–2021 (Appendix p3) [9, 10]. The prevalence, incidence, DALYs, and risk factors for RA were downloaded from publicly available GBD 2021, and all estimates were presented as count and age-standardized rate (ASR) per 100,000 population, with 95% uncertainty interval (UI) (https://vizhub.healthdata.org/gbd-results/).

### Case definition of RA

The 1987 American College of Rheumatology (ACR) classification criteria (Appendix p 3) were used as the reference diagnostic definition for RA in the GBD 2021 study [11]. Data sources that used diagnostic criteria other than the reference criteria (e.g., 2010 ACR-European League Against Rheumatism (EULAR) criteria, self-reported RA, or RA identified through administrative data) were adjusted with the MR-BRT (Meta-Regression Bayesian Regularized Trimmed). The research methodology used by the GBD has been described in the official GBD methodological appendix (https://www.healthdata.org/gbd/methods-appendices-2021) [12].

### Socio-demographic index

The socio-demographic index (SDI) is a composite indicator developed by GBD researchers to assess a country’s level of development based on the total fertility rate under the age of 25 (TFU25), mean education for those ages 15 and older (EDU15 +), and lag distributed income (LDI) per capita. It ranges from 0 (least developed) to 1 (most developed). Especially, for GBD 2021, the SDI values were multiplied by 100, ranging from 0 to 100, with higher scores indicating better socioeconomic conditions. The 204 countries and territories were then grouped into five SDI quintiles, including high, high-middle, middle, low-middle, and low (Appendix p 3) [13]. This study used the Pearson correlation coefficient and generalized additive models to explore the possible linear and non-linear relationship between the age-standardized prevalence rate (ASPR), age-standardized incidence rate (ASIR), age-standardized DALYs rate (ASDR), and the SDI, in order to better understand the influence of socioeconomic factors on the burden of RA.

### Estimates of risk factors attributable to RA

The GBD 2021 estimates the attributable burden of disease for 88 risk factors. Smoking is known to be a risk factor for RA and there is a correlation between them. We have obtained the percentage of RA DALYs attributable to smoking risk factors, which will be used to analyze the differences in the impact of smoking on RA burden across different regions (Appendix p 3) [14]. Further, the causal relationships between smoking (exposures) and RA (disease outcomes) were detected by two-sample Mendelian randomization analysis using genetic instruments as probes (Appendix p 4). The genome-wide association studies (GWAS) summary data for RA (ebi-a-GCST000679), smoking initiation (ieu-b-4877), and current tobacco smoking (ukb-b-223) were obtained from open gwas project (https://gwas.mrcieu.ac.uk/). According to the selection criteria of instrumental variables (IVs), 41 SNPs in the smoking initiation study and 13 SNPs in the current tobacco smoking study were used as IVs. (Appendix p 4) The inverse variance weighted (IVW) method was used as the standard for the results of the MR.

### Statistical analysis

#### Age-standardized rates

Age is an important factor affecting the burden of disease. In order to compare the burden of disease among different regions and countries, it is necessary to standardize the prevalence, incidence, and DALYs rates of diseases. ASR is a weighted average of age-specific (crude) rates per 100,000 population, which was used to quantify the level of the global burden of RA [15].

$$ASR= \frac{{\sum }_{i=1}^{A}{a}_{i}{w}_{i}}{{\sum }_{i=1}^{A}{a}_{i}}*\text{100,000}$$

Where ${a}_{i}$ is age-specific (crude) rate of the ${i}^{th}$ age group, and ${w}_{i}$ is the weight of individuals in the corresponding age group in the standard population.

#### Average annual percentage change

To assess the magnitude and direction of temporal trends in the prevalence, incidence, and DALYs of RA, we calculated average annual percentage change (AAPC) and corresponding 95% confidence interval (CI) by joinpoint regression (Appendix p 4) [16].

#### Bayesian age-period-cohort analysis

Age-period-cohort (APC) analysis was mainly used to analyze the changing trend of incidence of chronic diseases and to predict the future burden of disease. Factors considered include age, period, and cohort, but there is a linear relationship among the three, which may lead to non-unique parameter estimates. However, Bayesian APC (BAPC) uses sample information and prior information to obtain unique parameter estimates, and the obtained results are robust and reliable [17, 18]. In this study, projections of RA-related ASIR for 2022–2050 were generated using the BAPC package (R version 4.3.2).

### Patient and public involvement

We designed the study without considering involving patients because we used secondary data from the GBD 2021. No patients or members of the public were involved in the design, conduct, reporting, or dissemination plans of the study.

## Results

### The estimates of RA from 1990 to 2021

Overall, the global burden of RA continues to grow. In 2021, the estimated number of people (all ages) living with RA globally was 17.9 million (95% UI: 15,973,178 to 20,303,303), representing an increase of 125% (95% UI: 121% to 130%) compared to 1990. The global ASPR in 2021 was 208.9 (95% UI: 186.34 to 236.33) per 100,000 population, and the AAPC from 1990 to 2021 was 0.441 (95% CI: 0.419 to 0.464) (Table S1). The incidence cases for RA were 488,269 (95% UI: 435,015 to 545,895), with the ASIR of 11.8 (95% UI: 10.64 to 13.12) per 100,000 population, showing an increased trend in global ASIR (AAPC: 0.406, 95% CI: 0.388 to 0.424) (Table S1). RA resulted in 3,075,303 (95% UI: 2,310,381 to 3,974,046) global DALYs (all ages) in 2021, with the ASDR of 35.89 (95% UI: 26.95 to 46.46) per 100,000 population. There was no significant change in the global ASDR from 1990 to 2021 (AAPC: −0.046, 95% CI: −0.098 to 0.007) (Table S1, Figure S1).

In 2021, out of 21 GBD super regions, the ASPR and ASIR per 100,000 population of RA were highest in Andean Latin America (ASPR = 432.76, 95% UI: 384.44 to 486.44; ASIR = 21.72, 95% UI: 19.62 to 23.94), Central Latin America (ASPR = 360.24, 95% UI: 320.08 to 401.87; ASIR = 20.11, 95% UI: 18.11 to 22.31), and Australasia (ASPR = 354.82, 95% UI: 312.15 to 402.78; ASIR = 23.01, 95% UI: 20.53 to 25.58), and lowest in Oceania (ASPR = 50.75, 95% UI: 42.63 to 60.28; ASIR = 2.56, 95% UI: 2.2 to 2.97), Western Sub-Saharan Africa (ASPR = 61.79, 95% UI: 51.74 to 73.64; ASIR = 3.45, 95% UI: 2.99 to 3.95), and Southeast Asia (ASPR = 74.62, 95% UI: 64.01 to 87.73; ASIR = 4.23, 95% UI: 3.73 to 4.81) (Table S1, Figure S1). The Andean Latin America, Southern Latin America, and North Africa and Middle East show the largest increasing trend in ASPR and ASIR. In contrast, Southern Sub-Saharan Africa, High-income Asia Pacific, and Tropical Latin America were the only three regions to show a decreasing trend (Table S1, Figure S2). Regional differences in ASDR ranged from 68.34 (95% UI: 53.32 to 86.47) per 100,000 population in Central Latin America to 7.06 (95% UI: 4.6 to 10.45) in Oceania. From 1990 to 2021, the ASDR due to RA per 100,000 population increased the most in Central Asia (AAPC:1.45, 95% CI: 0.977 to 1.925), and decreased the most in High-income Asia Pacific (AAPC: −0.885, 95% CI: −1.032 to −0.737) (Table S1, Figure S1-S2).

**Fig. 1:**
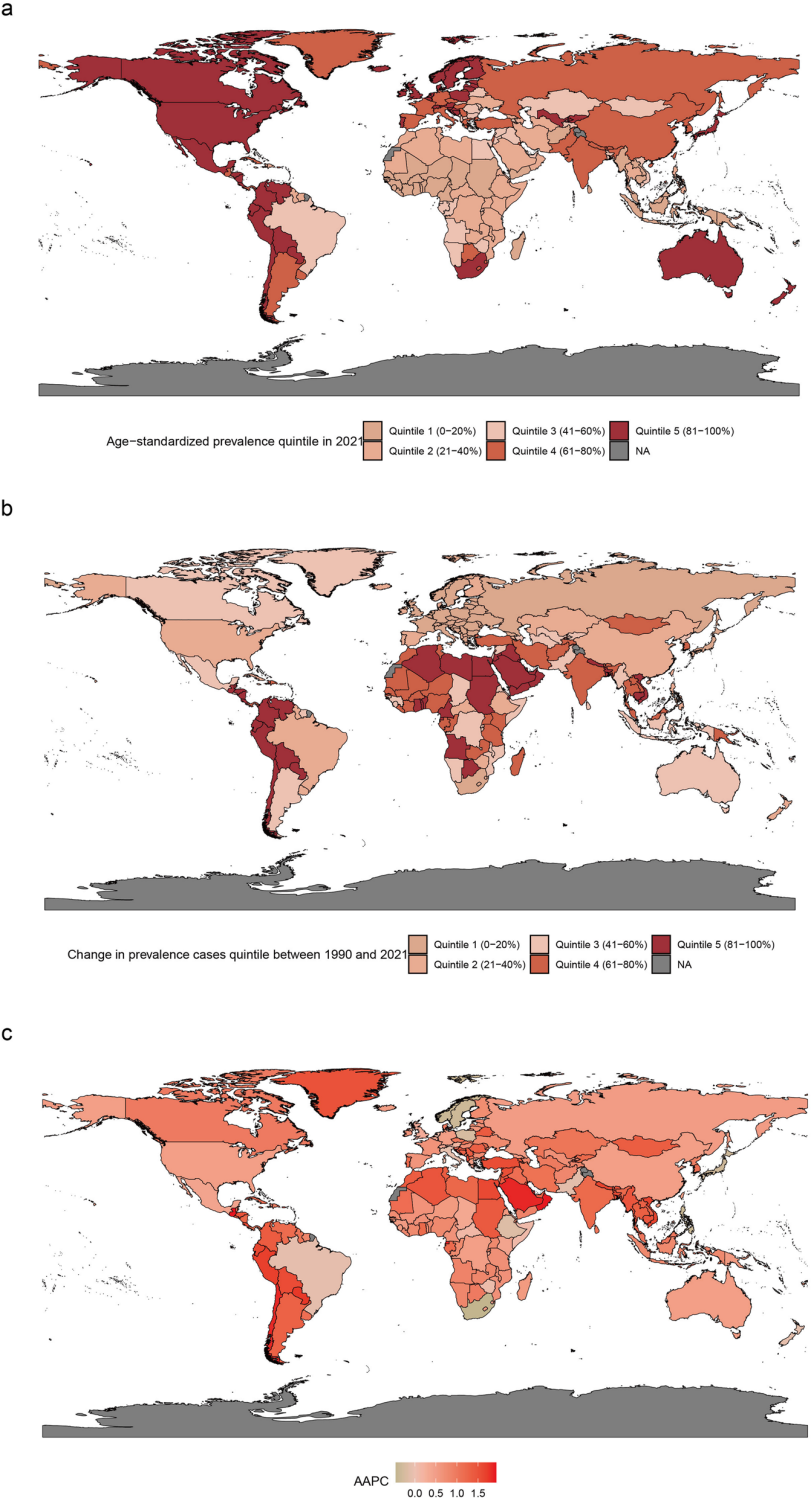
Global map of ASPR of RA categorized by ASPR quintiles for both sexes (**a**), change in prevalence cases quintiles for both sexes (**b**), and corresponding AAPC from 1990 to 2021 for both sexes (**c**). ASPR, age-standardized prevalence rate; RA, rheumatoid arthritis; AAPC, average annual percentage change

Among 204 countries and territories, the ASPR per 100,000 people of RA was highest in Ireland (539.08), Peru (520.62), and Finland (458.5) while Indonesia (46.19), Papua New Guinea (46.34) and Chad (48.88) showed the lowest rates (Fig. 1, Table S2). The ASIR per 100,000 people of RA was highest in Ireland (35.08, 95% UI: 31.79 to 38.82) and the largest increase in Oman (AAPC: 1.899, 95% CI: 1.859 to 1.939). Contrary, Papua New Guinea is the lowest in ASIR, and Norway (AAPC: −0.43, 95% CI: −0.472 to −0.388) showed the largest decreasing trend (Figure S3, Table S3). The top three countries with the highest ASDR were Mexico (87.45, 95% UI: 68.51 to 110.52), Ireland (82.49, 95% UI: 59.97 to 110.57) and Honduras (81.42, 95% UI: 63.33 to 105.19). Whereas, Papua New Guinea (6.49, 95% UI: 4.19 to 9.69), Chad (6.78, 95% UI: 4.55 to 9.69) and Kiribati (6.91, 95% UI: 4.47 to 9.98) showed the lowest rates. Mauritius (AAPC: 2.444, 95% CI: 1.073 to 3.833) recorded the most significant increase, whereas Japan (AAPC: −0.878, 95% CI: −1.038 to −0.718) saw the largest reduction (Figure S4, Table S4).

More information about prevalence, incidence, and DALYs at regional and national levels is provided in the Appendix (p5-7).

### The impact of SDI on the burden of RA

**Fig. 2:**
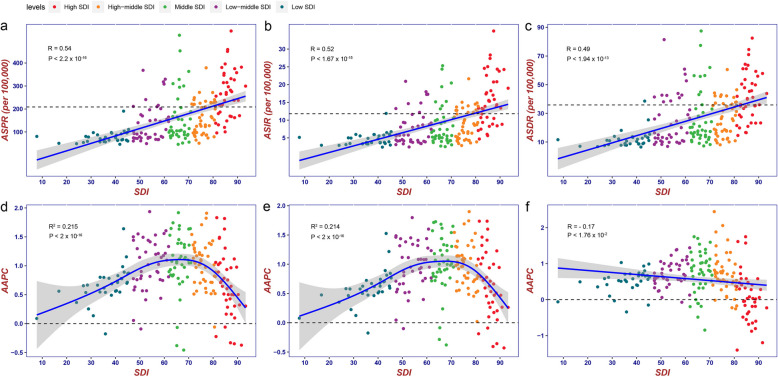
Association between SDI and ASPR (**a**), ASIR (**b**) and ASDR (**c**) of RA in 2021 and corresponding AAPC (**d**, **e**, **f**) from 1990 to 2021. Dotted lines refer to the global level of rates (**a**, **b**, **c**) and zero (**d**, **e**, **f**), respectively. SDI, socio-demographic index; ASPR, age-standardized prevalence rate; ASIR, age-standardized incidence rate; ASDR, age-standardized DALYs rate; DALYs, Disability-Adjusted Life Years; RA, rheumatoid arthritis; AAPC, average annual percentage change

The five levels of the SDI (Table S5-S6) show the association with ASRs (including ASPR, ASIR, and ASDR). ASPR ($R=0.54, P<2.2\times {10}^{-16}$), ASIR ($R=0.52, P<1.67\times {10}^{-15}$), and ASDR ($R=0.49, P<1.94\times {10}^{-13}$) are positively correlated with the SDI, which means that countries with higher SDI have correspondingly higher ASRs, while low SDI countries have lower ASRs (Fig. 2a-c). From 1990 to 2021, the ASPR, ASIR, and ASDR in high SDI region have been significantly higher than the global average level. Compared to other SDI regions, the high SDI region had the highest ASPR (282.73, 95% UI: 256.44 to 313.88), ASIR (16.88, 95% UI: 15.44 to 18.47) and ASDR (44.15, 95% UI: 32.56 to 58.11) in 2021 (Table S1). On the contrary, in the low SDI region, the ASPR, ASIR, and ASDR are the lowest among the five SDI-level regions, and they are significantly lower than the global average level. The burden of RA in high-middle SDI and middle SDI regions is relatively close to the global average level.

However, the association between the AAPC of ASPR ($R^{2}=0.215, P<2\times {10}^{-16}$) and ASIR with SDI ($R^{2}=0.214, P<2\times {10}^{-16}$) showed an inverted U-shaped curve (Fig. 2d-e) and the AAPC of ASDR ($R=-0.17, P<1.76\times {10}^{-2}$) was negatively correlated with the SDI (Fig. 2f). Most middle SDI countries have higher AAPC of ASPR and ASIR and low-middle SDI region showed the highest AAPC of ASRs (AAPC of ASPR: 1.069, 95% CI: 1.049 to 1.09; AAPC of ASIR: 0.934, 95% CI: 0.913 to 0.954; AAPC of ASDR: 0.559, 95% CI: 0.407 to 0.712). From 1990 to 2021, ASPR and ASIR rose across all SDI regions, while ASDR decreased in high SDI region (AAPC: −0.395, 95% CI: −0.46 to −0.33) (Table S1).

### Burden stratified by age and sex shows females are more affected by RA than males

**Fig. 3:**
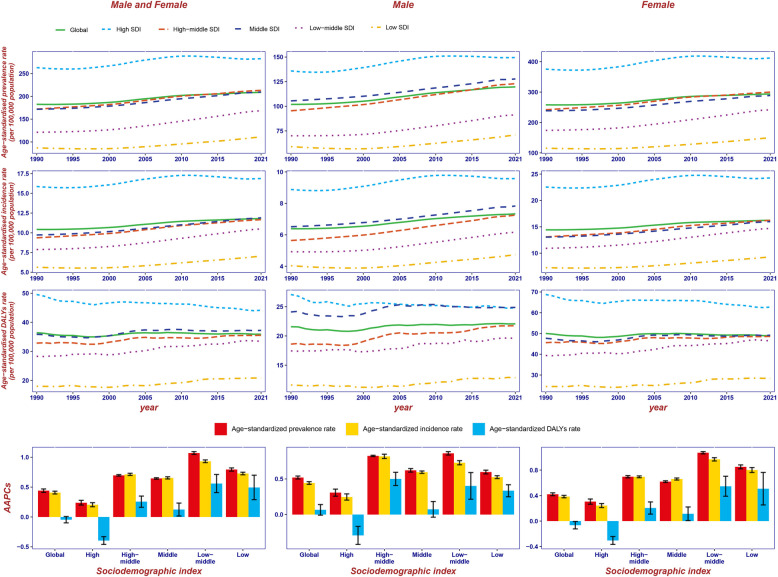
Temporal trend of ASPR, ASIR, and ASDR for the burden of RA, globally and by SDI (five levels: high, high-middle, middle, low-middle, and low SDI) from 1990 to 2021. The AAPC, globally and by SDI levels, from 1990 to 2021 is also shown. ASPR, age-standardized prevalence rate; ASIR, age-standardized incidence rate; ASDR, age-standardized DALYs rate; DALYs, Disability-Adjusted Life Years; RA, rheumatoid arthritis; SDI, socio-demographic index; AAPC, average annual percentage change

At the global level, the ASPR, ASIR, and ASDR were significantly higher in females than in males (Figure S5). The females had higher ASRs than males in five levels of SDI regions. In high SDI region, ASRs for males and females were higher than the global average level. Notably, in middle SDI region, ASRs for males are higher than the global average level, while those for females are below it (Fig. 3). For males, ASPR and ASIR were highest in the high SDI region and lowest in the low SDI region between 1990 and 2021. For males, prior to 2005, high SDI region had higher ASDR, but after that, the rates in middle and high SDI regions became similar and were both the highest. For females, ASRs were highest in the high SDI region, followed by high-middle and middle regions, which had similar rates, and lowest in the low SDI region.

**Fig. 4:**
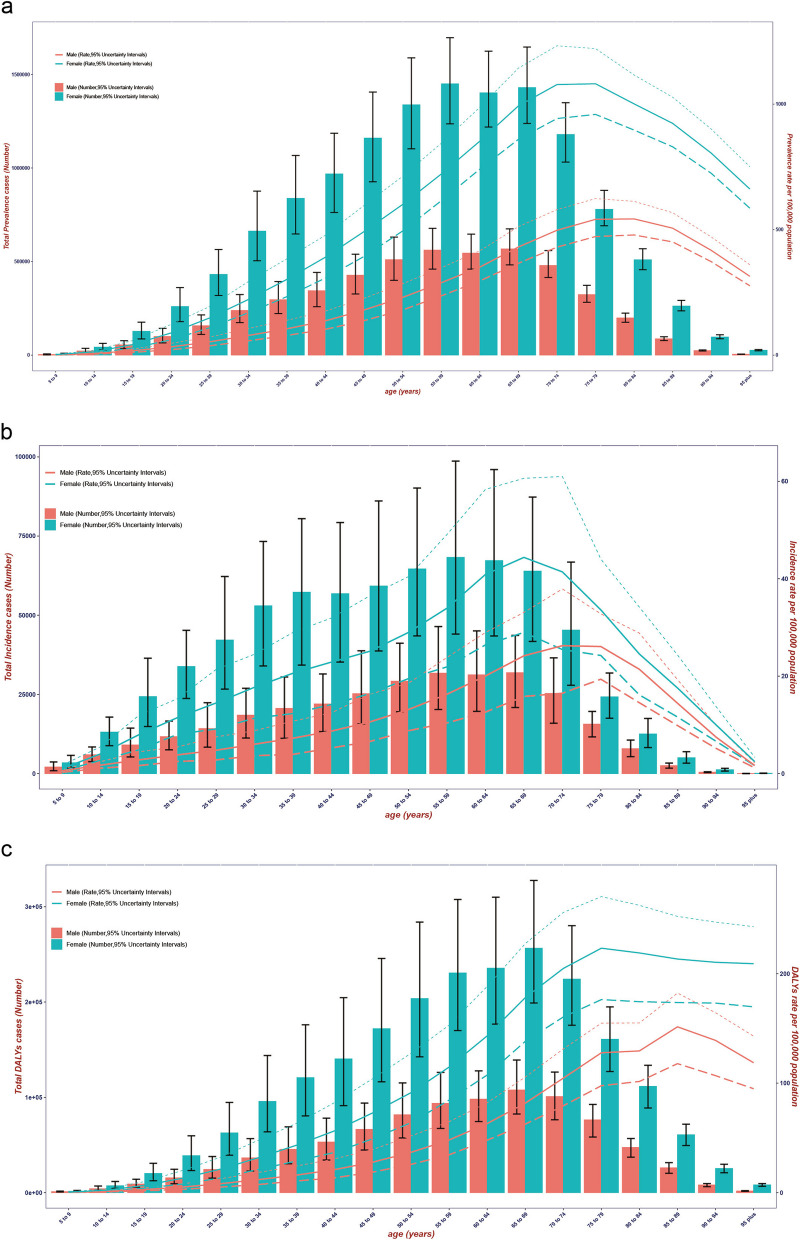
Global number of prevalent cases and age-specific prevalence rate (**a**), number of incidence cases and age-specific incidence rate (**b**), and DALYs and age-specific DALYs rate (**c**) of RA per 100,000 population by age and sex in 2021; dotted and dashed lines indicate 95% upper and lower uncertainty intervals, respectively. DALYs, Disability-Adjusted Life Years; RA, rheumatoid arthritis

The age-specific prevalence rate, incidence rate, and DALYs rate for females reached the peak at younger ages than in males, at 75–79 years, 65–69 years, and 75–79 years, respectively, whereas for males, the peaks occurred at 80–84 years, 70–74 years, and 85–89 years. The highest prevalence and incidence cases were observed among females aged 55–59 years and males aged 65–69 years, respectively, after which both showed a downward trend (Fig. 4a-b). DALYs reached the highest level in the 65–69 years age group for both females and males (Fig. 4c).

### Smoking is a causal risk factor for RA

In GBD 2021, smoking was the only risk factor for RA. The global percentage of DALYs attributed to RA due to smoking dropped from 9.43% in 1990 to 6.9% in 2021, showing a downward trend across all five SDI levels and higher in males than in females.

**Fig. 5:**
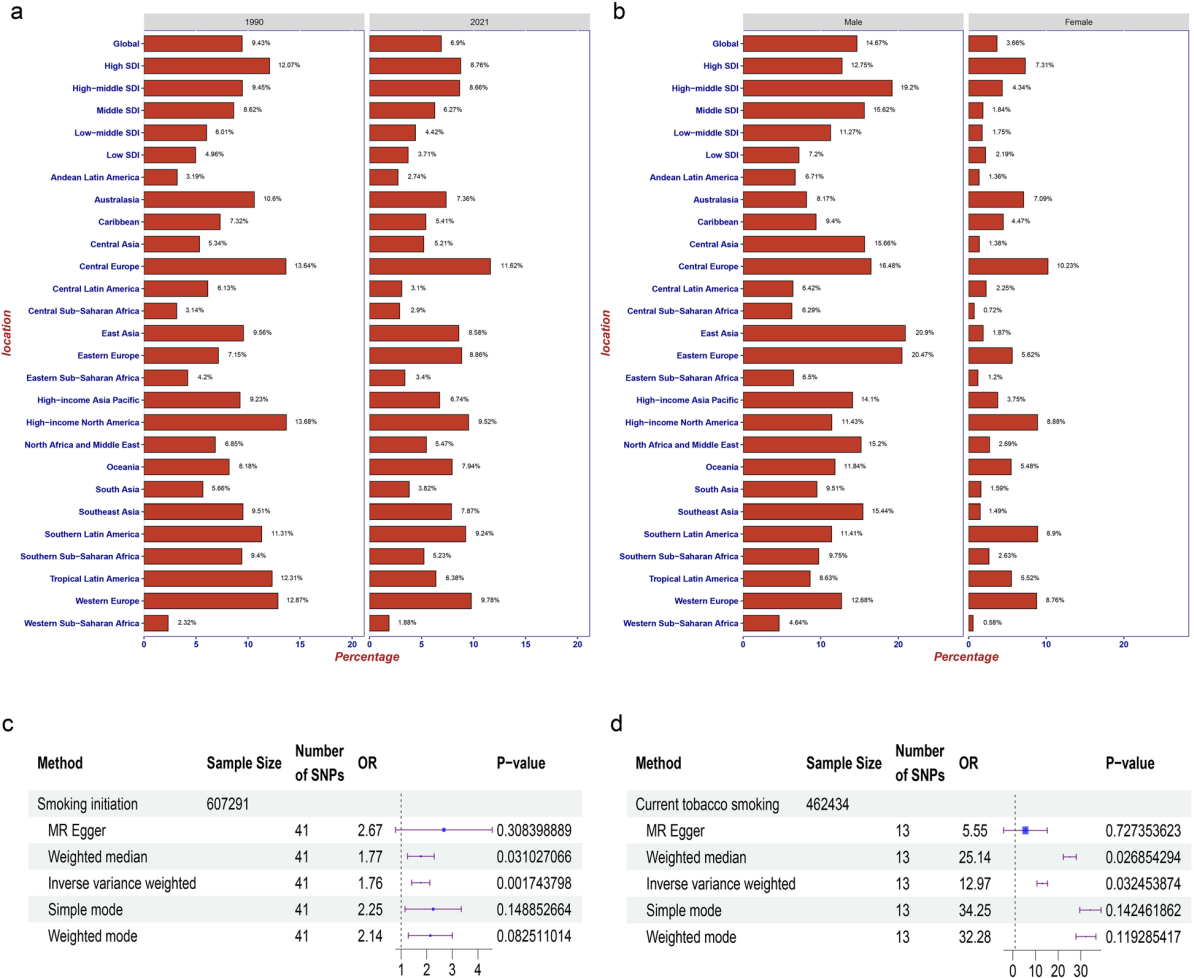
Proportion of RA DALYs attributable to smoking risk factor globally and in 26 GBD regions; combined in year (**a**) and sex (**b**), and forest plot of MR analysis combined in (**c**) the association of smoking initiation with risk of RA and (**d**) the association of current tobacco smoking with risk of RA. RA, rheumatoid arthritis; GBD, Global Burden of Disease Study; DALYs, Disability-Adjusted Life Years; MR, Mendelian randomization; OR, odds ratio; SNP, single nucleotide polymorphism

An analysis of the geographic area from 1990 to 2021 shows that Eastern Europe was the only region to experience a percentage increase from 7.15% to 8.86%, while Tropical Latin America exhibited a significant downward trend from 12.31% to 6.38%. In 2021, Central Europe (11.62%), Western Europe (9.78%), and High-income North America (9.52%) showed the highest percentages of DALYs attributable to smoking. Conversely, Western Sub-Saharan Africa (1.88%), Andean Latin America (2.74%), and Central Sub-Saharan Africa (2.9%) had the lowest percentages (Fig. 5a). The highest percentage of DALYs attributable to smoking was observed in East Asia for males and in Central Europe for females (Fig. 5b).

We further used MR analysis to evaluate the association between smoking and RA. The results of the two-sample MR analysis (Table S7 and Appendix p8) indicated that smoking initiation was significantly positively associated with the risk of RA compared with never-smokers (odds ratio (OR) = 1.76, 95% CI = 1.40–2.12, *P* < 0.05) using the IVW method (Fig. 5c). In addition, to verify whether this result was reproducible, we examined whether smoking and RA exhibited a similar causal relationship in the current tobacco smoking study. Similarly, current tobacco smoking studies show that current tobacco smoking was positively associated with increased risk of RA (OR = 12.97, 95% CI = 10.62–15.32, *P* < 0.05 for the IVW method) which further identification of causal associations between smoking and RA (Fig. 5d).

### Age-standardized incidence rate forecast by the Bayesian age-period-cohort model

**Fig. 6:**
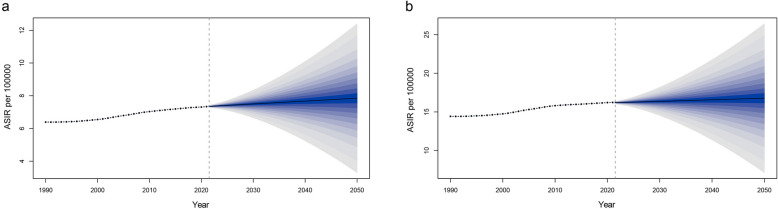
Global ASIR of RA from 1990 through 2050 forecasts in males (**a**) and females (**b**) predicted by BAPC model. The shaded area represents 95% CI. ASIR, age-standardized incidence rate; RA, rheumatoid arthritis; BAPC, Bayesian age-period-cohort; CI, confidence interval

The BAPC analysis showed that ASIRs for males and females would continue to increase at a slow speed with similar trends. By 2050, the ASIRs are expected to be around 7.85 per 100,000 population in males and 16.78 per 100,000 population in females (Fig. 6). Through the prediction of different age cohorts of males and females, we found that the changing trend of the age-specific incidence rates in each age group was roughly the same, which developed in a relatively stable trend. Across all age groups, the ASIR will be significantly higher in females than in males (Figure S6 and Figure S7).

## Discussion

RA is a significant burden in the field of global public health, but the current understanding of the burden of RA is insufficient. Previous studies were not comprehensive and in-depth because they focused on a certain field to analyze the global burden of RA. This study presents several key innovations that distinguish it from previous research on the global burden of RA. First, we utilize the most recent GBD 2021 data, providing updated insights into the global burden of RA through 2021. Second, our analysis offers a more comprehensive and RA-specific examination than prior studies that either focused on specific regions or grouped RA under broader disease categories like autoimmune or musculoskeletal diseases. By analyzing RA burden from global, regional, SDI, age, and gender perspectives, we offer a more nuanced understanding of how RA burden varies across different perspectives. Lastly, we go beyond previous GBD studies that merely listed smoking as a risk factor by applying MR analysis to explore and confirm the causal relationship between smoking and RA. Our study aims to provide the most comprehensive analysis to date by assessing the prevalence, incidence, and DALYs counts and ASRs for RA in 204 countries and territories from 1990 to 2021 based on GBD 2021. In 2021, there were 17.9 million prevalence cases, 1 million incidence cases, and 3.08 million DALYs worldwide.

We found that there are gender and age differences in the burden of RA. At the global and most regional levels, the ASR and the total number of cases were higher in females than in males. The burden is greater among middle-aged and elderly populations compared to adolescents. A number of studies have examined this sex difference, offering possible explanations such as hormonal factors, genetic factors, less access to treatment, and lower treatment effectiveness in females than in males [19, 20]. There were significant differences in the number of cases and age-specific rates among different age groups, but they all showed a trend of first increasing and then decreasing with age. It is worth noting that the peak of the number of cases and age-specific rates often did not occur in the same age group. This is usually due to differences in the proportion of the population in different age groups to the total population.

SDI is an important indicator to measure the level of socio-economic development of a country, which will have an impact on the burden of disease. Our result showed that ASPR, ASIR, and ASDR for both sexes varied from 1990 to 2021 globally and in all five SDI levels regions in Fig. 3. The disparities in ASRs across SDI regions highlight a clear link between socioeconomic status and RA burden. The high SDI region saw decreasing ASRs over the past decade, reflecting successful interventions, while the burden in other regions continues to rise. The low-middle SDI region had the highest AAPC in disease metrics, signaling a growing challenge. Notably, the high SDI region maintained the highest DALYs burden, particularly among females, while males experienced a greater burden in both high and middle SDI regions. These trends underscore the need for region-specific public health strategies. A previous study showed that countries with a higher SDI had a higher burden of RA [5, 21]. In our study, a positive correlation was also found between ASRs and SDI in RA (*P* < 0.05). This highlights the urgent need to investigate the determinants of RA burden and to establish effective prevention and treatment strategies in countries with high SDI.

It is known to all that environmental and genetic factors may be associated with RA. Here, we found smoking has the clearest association with RA by a disease burden analysis [22–24]. Globally, the number of DALYs attributable to smoking decreased by 2.53 percent from 1990 to 2021. It may be related to the downward trend in the global age-standardized daily smoking prevalence in both men and women [25]. Interestingly, females have higher ASDR than males, but the proportion of DALYs attributable to smoking is lower in females than males. This suggests that there are other risk factors that contribute to the burden of DALYs for RA, but the GBD 2021 currently only considers smoking as a risk factor. In the future, GBD 2021 should include more RA-related risk factors. One of the best ways to reduce DALYs caused by RA is to focus on risk factor management, especially in regions such as Western and Central Europe, where the proportion of DALYs attributable to smoking is high. The specific ways of smoking management can be found in previous relevant studies [26].

Smoking is the only RA risk factor included in GBD 2021, and epidemiological studies have found that smoking is positively correlated with the risk of RA [27, 28]. However, traditional observational studies are susceptible to biases such as confounding and reverse causality, making the causality behind this association ambiguous. Here, we applied the MR approach to investigate the potential causal relationship between smoking and RA. Two-sample MR analysis was performed using IVW as the final standard. The result of the two-sample MR analysis confirmed a causal association between smoking and increased risk of RA. Compared to never-smokers, genetic susceptibility to smoking initiation is associated with a 76% increased risk of RA. Furthermore, current tobacco smoking is significantly associated with a substantial increase in the risk of RA.

This study used the BAPC model to predict the ASIRs of males and females from 2022 to 2050. BAPC’s analysis shows that the incidence of RA will continue to increase at a low speed, with no fluctuations. This indicates that although RA still is a significant burden on global public health, it is generally stable and will not have a sudden explosive growth, which is conducive to the management and treatment of the burden.

Reducing the global burden of RA is still an urgent problem that needs to be solved worldwide. To reduce the global burden of RA, the first step is to strengthen the management of smoking, especially in those regions where the burden and the proportion of smoking-induced DALYs are higher. It takes a process from the onset of RA to the destruction of bones and joints [29]. So, early diagnosis is also very important to reduce the burden of RA [30]. Finally, regular follow-up is essential for RA patients in order to accurately monitor the progression of the disease and prevent bone and joint damage as much as possible [31].

This study also has several limitations. Firstly, the GBD 2021 data used in this study is based on extensive modeling estimates conducted by the GBD researchers. Despite their efforts to adjust for various potential influences, the conclusions drawn may still have certain biases compared to the actual burden in the real world. For instance, discrepancies in healthcare and diagnostic technologies across regions can lead to biases in the data sources, particularly in low- and middle-income regions. In these data-scarce areas, the accuracy of the model may still be a concern. Secondly, due to the global impact of the COVID-19 pandemic, inconsistent treatment protocols and timelines across countries make it difficult to ascertain whether the RA burden trends in 2021 were influenced by COVID-19. Thirdly, the GBD 2021 study lists smoking as the only risk factor for RA, but a previous study has shown that up to 40% of RA cases can be attributed to exposure to modifiable risk factors, including reducing inhalation of silica, dust, and occupational hazards, as well as avoiding high-salt diets [32]. Although targeting and managing these risk factors through specific interventions could reduce the burden of RA, these factors are not adequately discussed in the GBD study.

## Conclusion

RA is a growing health problem globally, especially in countries with high SDI. Females have a heavier burden of RA than males and need special attention to intervention measures. The ASPR, ASIR, and ASDR of RA all increased with age and began to decline after peaking in a certain age group. Over the past 32 years, smoking has been the only attributable risk factor for RA burden in all countries, and there is a significant causal association between them. From 2022 to 2050, the ASIR of RA globally will continue to increase at a low speed. Early diagnosis and treatment can help reduce the global burden of RA.

## Supplementary Information

Supplementary Material 1.

Supplementary Material 2.

## Data Availability

No datasets were generated or analysed during the current study.

## Abbreviations

RA: Rheumatoid arthritis
GBD: Global Burden of Disease Study
OR: Odds ratio
CI: Confidence interval
DALYs: Disability-Adjusted Life Years
SDI: Socio-demographic index
ASPR: Age-standardized prevalence rate
ASIR: Age-standardized incidence rate
ASDR: Age-standardized DALYs rate
AAPC: Average annual percentage change
MR: Mendelian randomization
SNP: Single nucleotide polymorphism
BAPC: Bayesian age-period-cohort
ASR: Age-standardized rate
UI: Uncertainty interval
