# Supplementary material for: Global, regional and national burden of rheumatoid arthritis from 1990 to 2021, with projections of incidence to 2050: a systematic and comprehensive analysis of the Global Burden of Disease study 2021
Source: Biomark Res. 2025 Mar 24;13:47. doi: 10.1186/s40364-025-00760-8 (PMC11931880; doi:10.1186/s40364-025-00760-8)
Supplement: Supplementary file 1 — Supplementary Material 1. [file 40364_2025_760_MOESM1_ESM.docx]

**Supplementary Appendix**

**Global, regional and national burden of rheumatoid arthritis from 1990-2021, with projections of incidence to 2050: a systematic and comprehensive analysis of the Global Burden of Disease study 2021**

**[Table of content](#bookmark1)**

[Section 1. Supplementary Methods 2](#bookmark2)

[Data Source 2](#bookmark3)

[Case definition of RA 3](#bookmark4)

[Socio-demographic Index (SDI) 3](#bookmark5)

[Estimates of risk factors attributable to RA from the GBD 2021 3](#bookmark6)

[Joinpoint 3](#bookmark7)

[Two-sample Mendelian randomization 3](#bookmark8)

[Reference 4](#bookmark9)

[Section 2. Supplementary Results 4](#bookmark10)

[Analysis of global burden from 1990 to 2021 4](#bookmark11)

[Analysis of regional burden from 1990 to 2021 5](#bookmark12)

[Analysis of national burden from 1990 to 2021 6](#bookmark13)

[Smoking is associated with the risk for RA 7](#bookmark14)

[Section 3. Supplementary Figures 8](#bookmark15)

[Figure S1 8](#bookmark16)

[Figure S2 9](#bookmark17)

[Figure S3 10](#bookmark18)

[Figure S4 11](#bookmark19)

[Figure S5 12](#bookmark20)

[Figure S6 13](#bookmark21)

[Figure S7 14](#bookmark22)

**Section 1. Supplementary Methods**

**Data Source**

All data used in the study were extracted from GBD 2021 (<https://vizhub.healthdata.org/gbd-results/>), including (1) global age-, sex- specific and standardized prevalence, incidence, DALYs counts and rates (per 100,000 population) from 1990 to 2021; (2) regional age-, sex- specific and standardized prevalence, incidence, DALYs counts and rates (per 100,000 population) from 1990 to 2021 in 21 GBD super regions and 5 SDI regions; (3) national age-, sex- specific and standardized prevalence, incidence, DALYs counts and rates (per 100,000 population) from 1990 to 2021 in 204 countries and territories;

(4) Regional and sex- specific RA DALYs rates attributable to risk factors (level 4); (5) GBD world

standard population in 2021; (6) GBD Estimate population from 1990 to 2021; (7) Population prediction 2018-2100; (8) GBD 2021 SDI 1950-2021.

**Case definition of RA**

The revised RA diagnostic criteria were developed through a computer analysis of 262 contemporary, continuous study patients with RA and 262 non-RA control patients. The seven diagnostic criteria (1. morning stiffness; 2. arthritis of three or more joint areas; 3. symmetric arthritis; 4. arthritis of hand joints; 5. rheumatoid nodules; 6. serum rheumatoid factor; 7. radiographical changes). The patients are diagnosed with RA when they have four or more symptoms. The first four criteria must persist for at least 6 weeks to fulfill the case definition.[1](#bookmark24)

**Socio-demographic Index (SDI)**

SDI is a comprehensive indicator of lagged distribution income per capita in a country, this includes gross domestic product per capita over the past 10 years, the average years of schooling for those over 15, and the total fertility rate for those under 25. It ranges from 0 (least developed) to 1 (most developed).[2](#bookmark25) In GBD 2021, SDI can be subdivided into five levels: Low SDI [0-0.466); Low-middle SDI [0.466-0.619); Middle SDI [0.619-0.712); High-middle SDI [0.712-0.810); High SDI [0.810-1] (Table S5). According to the above criteria, 204 countries and territories corresponded to their respective SDI levels (Table S6).

**Estimates of risk factors attributable to RA from the GBD 2021**

DALYs are the main manifestation of the burden of RA, and it may be caused by a variety of risk factors. The GBD 2021 estimates the attributable burden of disease for 88 risk factors, as well as a combination of risk factors at the global, regional, and national levels. Attributable DALY means that current disease DALY may be reduced if exposure to risk factors at the population level has shifted to another or counterfactual risk distribution.[3](#bookmark26)

**Joinpoint**

Joinpoint is statistical software for the analysis of trends using joinpoint models, which take trend data and fit the simplest joinpoint model that the data allows. The minimum and maximum number of connection points are provided by the users. The software performs a significance test by Monte Carlo Permutation, which enables the user to test whether an apparent change in trend is statistically significant[. 4](#bookmark27) *P* < 0.05 was considered as the statistical significance threshold.

**Two-sample Mendelian randomization**

Mendelian randomization, using genetic instruments as probes to detect causal relationships between exposures and disease outcomes, is perceived as a new study design that can minimize the risk of

confounding bias and reverse causation. The causal relationship between RA and smoking was explored using a Mendelian randomization analysis. The genome-wide association studies (GWAS) summary data for RA were obtained from open gwas project (ebi-a-GCST000679), including 5,539 RA cases and 20, 169 controls of European ancestry.[5](#bookmark28) The GWAS summary data for smoking initiation (ieu-b-4877) contains 607,291 individuals and the GWAS summary data of current tobacco smoking (ukb-b-223) were from UK biobank, including 462,434 individuals of European ancestry, respectively.[6](#bookmark29) The instrumental variables (IVs) were selected based on the following criteria: (1) conditionally independent single nucleotide polymorphisms (SNPs) associated with smoking at genome-wide significance threshold (P < 5 × 10− 8) were selected as potential instrumental variables (IVs); (2) The 1000 Genomes Project European samples are as a reference panel, to calculate linkage disequilibrium (LD) between SNPs. Among SNPs with r2 < 0.001 (with a window size of 10,000 kb), only the SNPs with the lowest p-value were retained. After removing SNPs that were not available in summary statistics data for RA, 41 IVs were identified in the smoking initiation study and 13 IVs in the current tobacco smoking study.

**Reference**

1. Arnett FC, Edworthy SM, Bloch DA, et al. The American Rheumatism Association 1987 revised criteria for the classification of rheumatoid arthritis. *Arthritis and rheumatism* 1988; **31**(3): 315-24.

2. Global burden of 369 diseases and injuries in 204 countries and territories, 1990-2019: a systematic analysis for the Global Burden of Disease Study 2019. *Lancet (London, England)* 2020; **396**(10258): 1204-22.

3. Global burden and strength of evidence for 88 risk factors in 204 countries and 811 subnational locations, 1990-2021: a systematic analysis for the Global Burden of Disease Study 2021. *Lancet* *(London, England)* 2024; **403**(10440): 2162-203.

4. Kim HJ, Fay MP, Feuer EJ, Midthune DN. Permutation tests for joinpoint regression with applications to cancer rates. *Statistics in medicine* 2000; **19**(3): 335-51.

5. Stahl EA, Raychaudhuri S, Remmers EF, et al. Genome-wide association study meta-analysis identifies seven new rheumatoid arthritis risk loci. *Nature genetics* 2010; **42**(6): 508-14.

6. Liu M, Jiang Y, Wedow R, et al. Association studies of up to 1.2 million individuals yield new insights into the genetic etiology of tobacco and alcohol use. *Nature genetics* 2019; **51**(2): 237-44.

**Section 2. Supplementary Results**

**Analysis of global burden from 1990 to 2021**

*Prevalence*

In 1990, there were 7,959,055 (95% UI: 7041,419 to 9085,469) prevalence cases of RA worldwide, and in 2021, the number of prevalence cases was 17,924,667 (95% UI: 15,973, 178 to 20,303,303), an increase of 125% (95% UI: 121% to 130%) compared with 1990. And global ASPR in 1990 was 182.54 (95% UI: 161.59 to 207.48), the rate in 2021 was 208.9 (95% UI: 186.34 to 236.33), and the AAPC from 1990 to 2021 was 0.441 (95% CI: 0.419 to 0.464) (Table S1).

*Incidence*

In 1990, there were 488,269 (95% UI: 435,015 to 545,895) incidence cases of RA around the world, and the number of incidence cases in 2021 was 1,000,319 (95% UI: 902,687 to 1, 114,213), an increase of 105% (95% UI: 98% to 111%) compared with 1990. And global ASIR in 1990 was 10.42 (95% UI: 9.32 to 11.64), the rate in 2021 was 11.8 (95% UI: 10.64 to 13. 12), and the AAPC from 1990 to 2021 was 0.406 (95% CI: 0.388 to 0.424) (Table S1).

*DALYs*

In 1990, there were 1,545,699 (95% UI: 1,201,479 to1,977,786) DALYs of RA at the global level, and the DALYs in 2021 were 3,075,303 (95% UI: 2,310,381 to 3,974,046), an increase of 99% (95% UI: 90% to 106%). And global ASDR in 1990 was 36.42 (95% UI: 28.71 to 46), and the rate in 2021 was 35.89 (95% UI: 26.95 to 46.46) (Table S1). The overall trend of DALYs was relatively stable from 1990 to 2021, but there was a significant downward trend from 2004 to 2021.

**Analysis of regional burden from 1990 to 2021**

*Prevalence*

At the regional level, the top three regions with the highest ASPRs in 2021 were Andean Latin America (432.76 (95% UI: 384.44 to 486.44)), Central Latin America (360.24 (95% UI: 320.08 to 401.87)), and Australasia (354.82 (95% UI: 312.15 to 402.78)). In contrast, the top three regions with the lowest ASPRs were Oceania (50.75 (95% UI: 42.63 to 60.28)), Western Sub-Saharan Africa (61.79 (95% UI:

51.74 to 73.64)), and Southeast Asia (74.62 (95% UI: 64.01 to 87.73)) (Table S1 and Figure S1a).

Between 1990 and 2021, prevalence cases increased in all regions of the world. The region with the largest percentage change is Andean Latin America (292% (95% UI: 269% to 314%)), followed by North Africa and Middle East (279% (95% UI: 264% to 295%)) and South Asia (227% (95% UI: 217% to 237%)) (Table S1).

From 1990 to 2021, the largest increase in ASPR of RA was observed in Andean Latin America (AAPC: 1.637, 95% CI: 1.591 to 1.682), followed by Southern Latin America (AAPC: 1.592, 95% CI: 1.572 to 1.612) and North Africa and Middle East (AAPC: 1.378, 95% CI: 1.355 to 1.401). In contrast, Southern Sub-Saharan Africa (AAPC: -0.32, 95% CI: -0.331 to -0.308), High-income Asia Pacific (AAPC: -0.249, 95% CI: -0.289 to -0.209), and Tropical Latin America (AAPC: -0.004, 95% CI: -0.059 to 0.05) were the only three regions to show the decreasing trend (Table S1 and Figure S2a).

*Incidence*

The top three regions with the highest ASIRs were also Australasia (23.01 (95% UI: 20.53 to 25.58)), Andean Latin America (21.72 (95% UI: 19.62 to 23.94)) and Central Latin America (20. 11 (95% UI: 18. 11 to 22.31)); whereas, Oceania (2.56 (95% UI: 2.2 to 2.97)), Western Sub-Saharan Africa (3.45 (95% UI: 2.99 to 3.95)) and Southeast Asia (4.23 (95% UI: 3.73 to 4.81)) showed the lowest rates (Table S1 and Figure S1b).

Between 1990 and 2021, incidence cases increased in all regions of the world. Andean Latin America (230% (95% UI: 208% to 250%)), Western Sub-Saharan Africa (220% (95% UI: 212% to 229%)) and Central Sub-Saharan Africa (219% (95% UI: 207% to 230%)) were the top three regions with the largest increases (Table S1).

From 1990 to 2021, the top three regions with the largest increase in ASIR were Andean Latin America (AAPC: 1.531, 95% CI: 1.474 to 1.589), Southern Latin America (AAPC: 1.451, 95% CI: 1.43 to 1.472) and North Africa and Middle East (AAPC:1.259, 95% CI: 1.234 to 1.284). Whereas, High-income Asia Pacific (AAPC: -0. -0.257), Southern Sub-Saharan Africa

29, 95% CI: -0.323 to

5

(AAPC: -0.29, 95% CI: -0.312 to -0.268), and Tropical Latin America (AAPC: -0.003, 95% CI: -0.061 to 0.056) were the only three regions showed the decreasing trend in ASIR (Table S1 and Figure S2b). *DALYs*

Central Latin America (68.34 (95% UI: 53.32 to 86.47)), Andean Latin America (67.82 (95% UI: 48.77 to 89.56)), and Australasia (54.8 (95% UI: 39.98 to 72.57)) were the top three regions with the highest ASDR. In contrast, the region with the lowest ASDR was Oceania (7.06 (95% UI: 4.6 to 10.45)), followed by Western Sub-Saharan Africa (8.48 (95% UI: 5.68 to 12.04)) and Eastern Sub-Saharan Africa (11.96 (95% UI: 8.16 to 17.28)) (Table S1and Figure S1c).

Between 1990 and 2021, DALYs increased in all regions of the world. Andean Latin America (237% (95% UI: 209% to 267%)) was the region with the largest percentage change in DALYs, followed by North Africa and Middle East (222% (95% UI: 194% to 248%)) and Western Sub-Saharan Africa (218% (95% UI: 206% to 231%)) (Table S1).

The top three increasing trends in ASDRs belonged to Central Asia (AAPC:1.45, 95% CI: 0.977 to 1.925), Andean Latin America (AAPC: 0.956, 95% CI: 0.722 to 1. 191), and Southern Latin America (AAPC: 0.854, 95% CI: 0.579 to 1. 129). Whereas, High-income Asia Pacific (AAPC: -0.885, 95% CI: -1.032 to -0.737) showed the largest decreasing trend, followed by Southern Sub-Saharan Africa (AAPC: -0.659, 95% CI: -0.874 to -0.444) and Central Europe (AAPC: -0.316, 95% CI: -0.449 to -0. 183) (Table S1 and Figure S2c).

**Analysis of national burden from 1990 to 2021**

*Prevalence*

In 2021, the ASPR of RA ranged from 46 to 539 cases per 100,000 population in 204 countries and territories worldwide. Ireland (539.08 (95% UI: 481.75 to 605. 19)), Peru (520.62 (95% UI: 457.53 to 587.32)) and Finland (458.5 (95% UI: 414.25 to 513. 17)) had the three highest ASPRs. Whereas, Indonesia (46.19 (95% UI: 38.7 to 55.4)), Papua New Guinea (46.34 (95% UI: 38.71 to 54.9)) and Chad (48.88 (95% UI: 41. 12 to 58.24)) showed the lowest rates (Table S2 and Figure 1a).

The increase in the relative change in the prevalence cases of RA between 1990 and 2021 was most noticeable in Qatar (1354% (95% UI: 1,226% to 1,510%)), followed by United Arab Emirates (1, 146% (95% UI: 1,027% to 1,300%)) and Jordan (638% (95% UI: 584% to 704%)). It is worth noting that all three countries are from North Africa and the Middle East region, a region experiencing the second-highest increase in the number of cases globally, with a significant burden of RA. In contrast, the three countries with the lowest relative growth were Georgia (-8% (95% UI: -14% to -2%)), Latvia (7% (95% UI: 0% to 15%)) and Lithuania (18% (95% UI: 10% to 26%)). Georgia is the only country in the world with fewer cases in 2021 than in 1990 (Table S2 and Figure 1b).

From 1990 to 2021, the three countries with the highest AAPC ofASPR were Guatemala (1.936 (95% CI: 1.909 to 1.963)), Equatorial Guinea (1.918 (95% CI: 1.865 to 1.971)) and Oman (1.909 (95% CI: 1.874 to 1.944)). South Africa (-0.457 (95% CI: -0.478 to -0.436)), Philippines (-0.38 (95% CI: -0.467 to -0.292)) and Norway (-0.372 (95% CI: -0.4 to -0.345)) had the most obvious decreasing trends in ASPR (Table S2 and Figure 1c).

*Incidence*

In 2021, ASIR varied from 2 to 35 cases per 100,000 population in 204 countries and territories worldwide. Ireland (35.08 (95% UI: 31.79 to 38.82)), Finland (28.33 (95% UI: 25.67 to 31.29)) and United Kingdom (27.37 (95% UI: 24.58 to 30.42)) were the three countries with the highest ASIRs in the world. All three countrie of RA ASIR is at a relatively

s are from Western Europe, where the burden

6

high level globally. Whereas, Papua New Guinea (2.39 (95% UI: 2.04 to 2.77)), Kiribati (2.43 (95% UI: 2.08 to 2.85)) and Fiji (2.52 (95% UI: 2. 1 to 3.05)) had the lowest rates in 2021. All three countries are located in Oceania, which is also the region with the lowest burden of RA ASIR globally (Table S3 and Figure S3a).

The magnitude of increase in the relative change in the incidence cases of RA between 1990 and 2021 was also most noticeable in Qatar (1190% (95% UI: 1058% to 1319%)), followed by United Arab Emirates (811% (95% UI: 643% to 987%)) and Bahrain (512% (95% UI: 427% to 607%)). Similar to the magnitude of increases in prevalence cases, Qatar and United Arab Emirates also had the highest increases in incidence. In contrast, Georgia (-20% (95% UI: -25% to -14%)), Latvia (-10% (95% UI: -16% to -4%)) and Lithuania (-6% (95% UI: -12% to 2%)) showed the decreasing situation. They are also the only three countries globally showing a declining trend (Table S3 and Figure S3b).

From 1990 to 2021, Oman (AAPC: 1.899 (95% CI: 1.859 to 1.939)), Guatemala (AAPC: 1.797 (95% CI: 1.749 to 1.845)), and Chile (AAPC: 1.742 (95% CI: 1.716 to 1.768)) had the highest increasing trend. Whereas, Norway (AAPC: -0.43 (95% CI: -0.472 to -0.388)), Japan (AAPC: -0.406 (95% CI: -0.467 to -0.345)) and South Africa (AAPC: -0.379 (95% CI: -0.398 to -0.361)) showed the decreasing trend. Of note, Norway and South Africa mentioned above similarly demonstrated the most noticeable decreasing trends in the annual change in ASPR (Table S3 and Figure S3c).

*DALYs*

The top three countries with the highest ASDR were Mexico (87.45 (95% UI: 68.51 to 110.52)), Ireland (82.49 (95% UI: 59.97 to 110.57)) and Honduras (81.42 (95% UI: 63.33 to 105. 19)). Whereas, Papua New Guinea (6.49 (95% UI: 4.19 to 9.69)), Chad (6.78 (95% UI: 4.55 to 9.69)) and Kiribati (6.91 (95% UI: 4.47 to 9.98)) showed the lowest rates (Table S4 and Figure S4a).

The most noticeable increase of DALYs was observed in Qatar (1300% (95% UI: 1077% to 1548%)), followed by United Arab Emirates (939% (95% UI: 764% to 1145%)) and Bahrain (665% (95% UI: 548% to 812%)). These countries also rank relatively high globally in terms of both the increase in prevalence and incidence cases (Table S4 and Figure S4b).

From 1990 to 2021, Mauritius (AAPC: 2.444 (95% CI: 1.073 to 3.833)), Bahrain (AAPC: 2.058 (95% CI: 1.872 to 2.244)) and Kazakhstan (AAPC: 1.88 (95% CI: 1.087 to 2.68)) had the largest increase in ASDRs. In contrast, Poland (AAPC: -1.402 (95% CI: -1.636 to -1. 168)), Norway (AAPC:

-1.197 (95% CI: -1.46 to -0.932)) and Japan (AAPC: -0.878 (95% CI: -1.038 to -0.718)) showed the most obvious decreasing trends (Table S4 and Figure S4c).

**Smoking is associated with the risk for RA.**

According to the selection criteria of IVs, 41 SNPs in the smoking initiation study and 13 SNPs in the current tobacco smoking study were used as IVs. In the smoking initiation study, the weighted median method provided a similar effect estimate to the IVW method (OR = 1.77, 95% CI = 1.25 - 2.91, *P* < 0.05). MR-Egger regression analysis indicated that there was no horizontal gene pleiotropy among the IVs in the smoking initiation study (intercept = -0.01, *P* = 0.66). In the current tobacco smoking study, the weighted median method provided a consistent effect estimate (OR =25. 14, 95% CI = 22.29 - 27.1, *P* < 0.05). There was no evidence for horizontal gene pleiotropy (intercept = 0.006, *P* = 0.86).

**Section 3. Supplementary Figures**


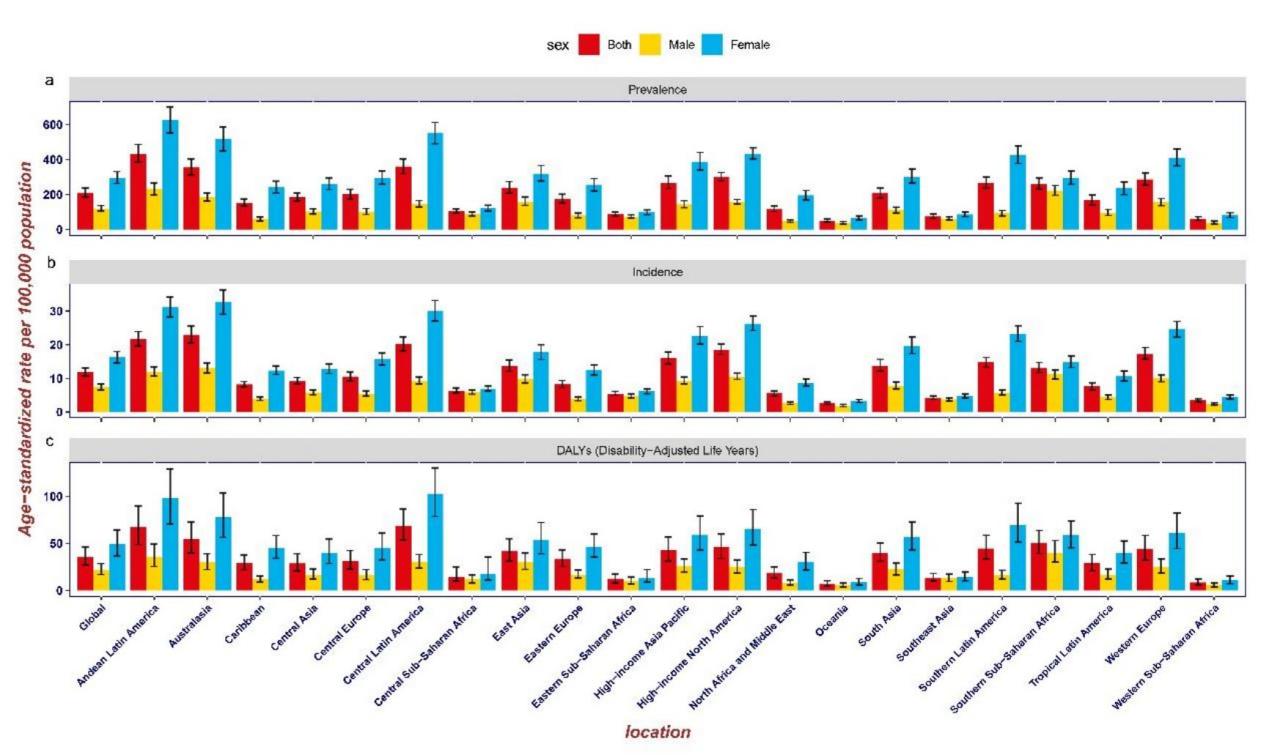


**Figure S1**. Difference of RA burden globally and in 21 GBD regions combine in ASPR per 100,000 population (a), ASIR per 100,000 population (b), and ASDR per 100,000 population (c) by sexes in 2021. RA, rheumatoid arthritis; GBD, Global Burden of Disease Study; ASPR, age-standardized prevalence rate; ASIR, age-standardized incidence rate; ASDR, age-standardized DALYs rate; DALYs, Disability-Adjusted Life Years.


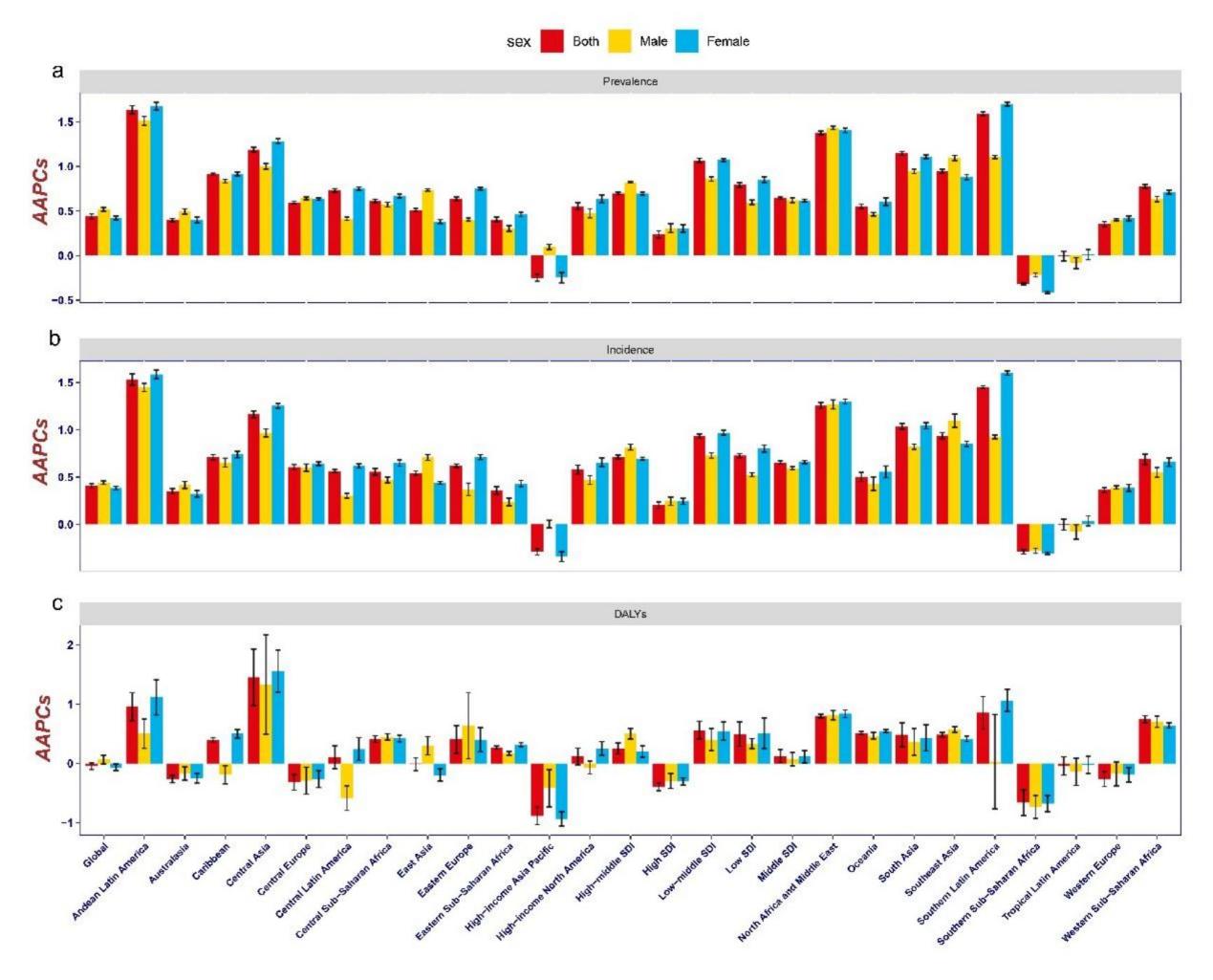


**Figure S2**. Difference of RA AAPCs globally and in 21 GBD regions combined in ASPR per 100,000 population (a), ASIR per 100,000 population (b), and ASDR per 100,000 population (c) by sexes from 1990 to 2021. RA, rheumatoid arthritis; AAPC, average annual percentage change; GBD, Global Burden of Disease Study; ASPR, age-standardized prevalence rate; ASIR, age-standardized incidence rate; ASDR, age-standardized DALYs rate; DALYs, Disability-Adjusted Life Years.


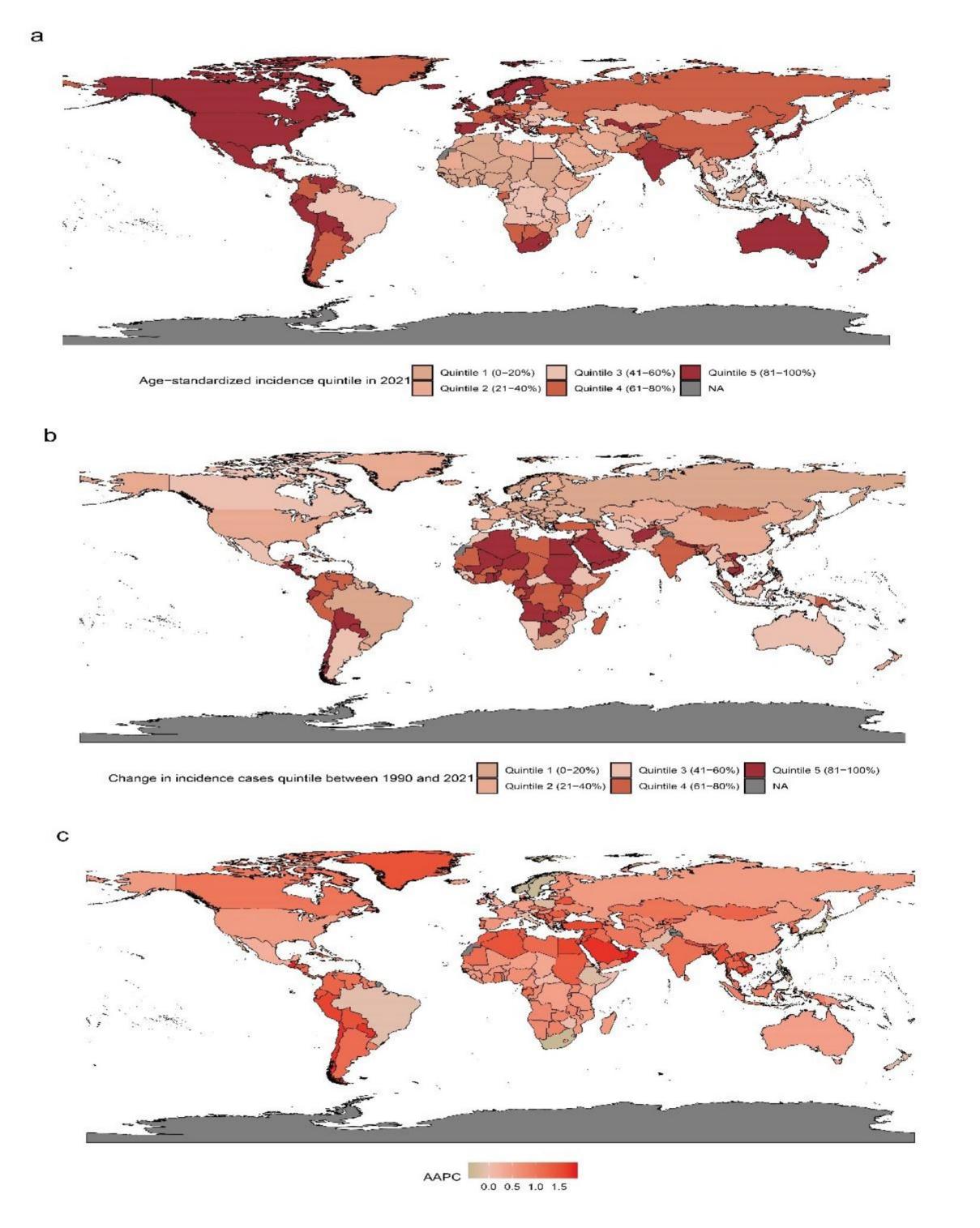


**Figure S3**. Global map of ASIRs of RA categorized by ASIR quintiles for both sexes (a), change in incidence cases quintiles for both sexes (b), and corresponding AAPC from 1990 to 2021 for both sexes (c). ASIR, age-standardized incidence rate; RA, Rheumatoid arthritis; AAPC, average annual percentage change.


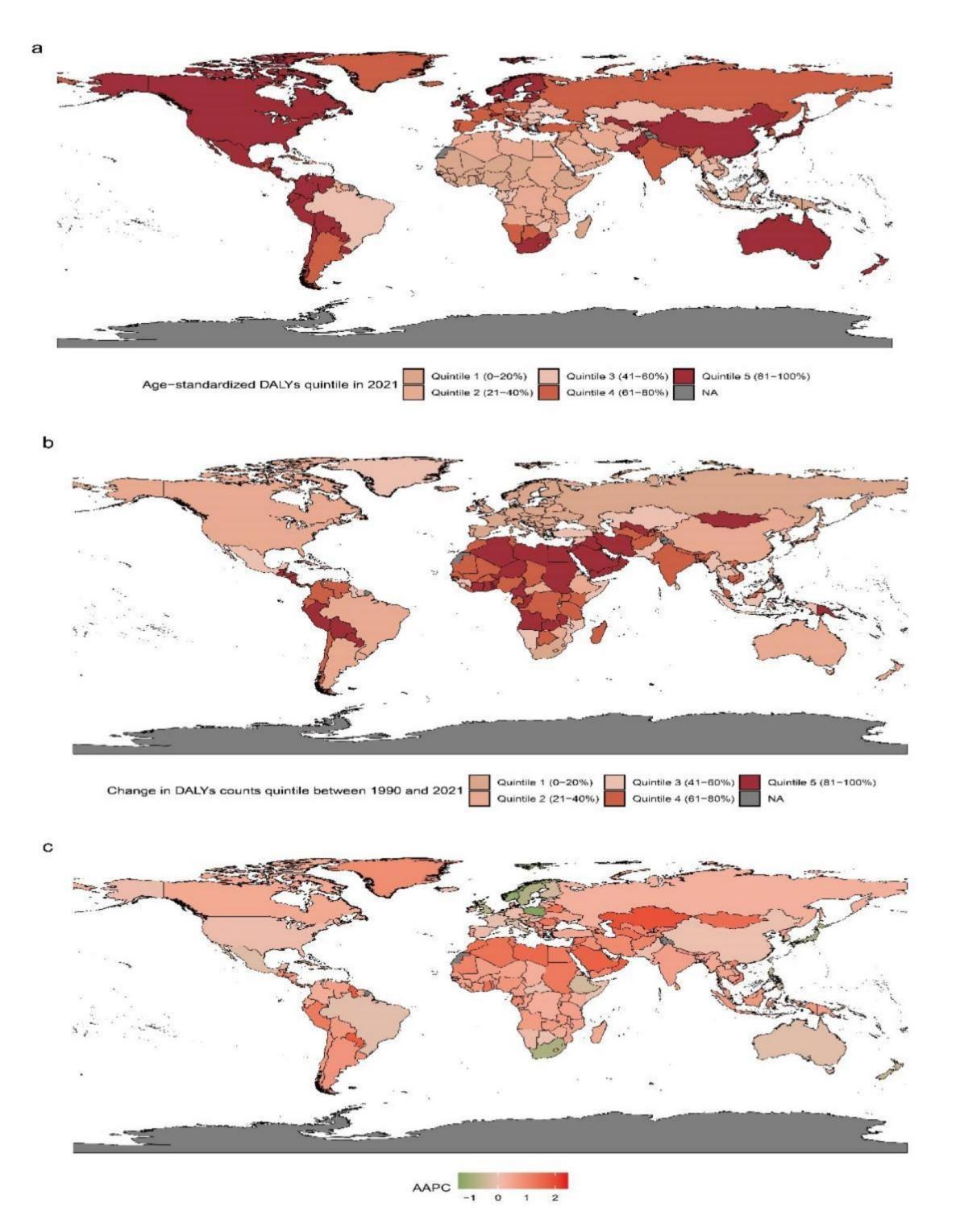


**Figure S4**. Global map of ASDRs of RA categorized by ASDR quintiles for both sexes (a), change in DALYs counts quintiles for both sexes (b), and corresponding AAPC from 1990 to 2021 for both sexes (c). ASDR, age-standardized DALYs rate; DALYs, Disability-Adjusted Life Years; RA, Rheumatoid arthritis; AAPC, average annual percentage change.


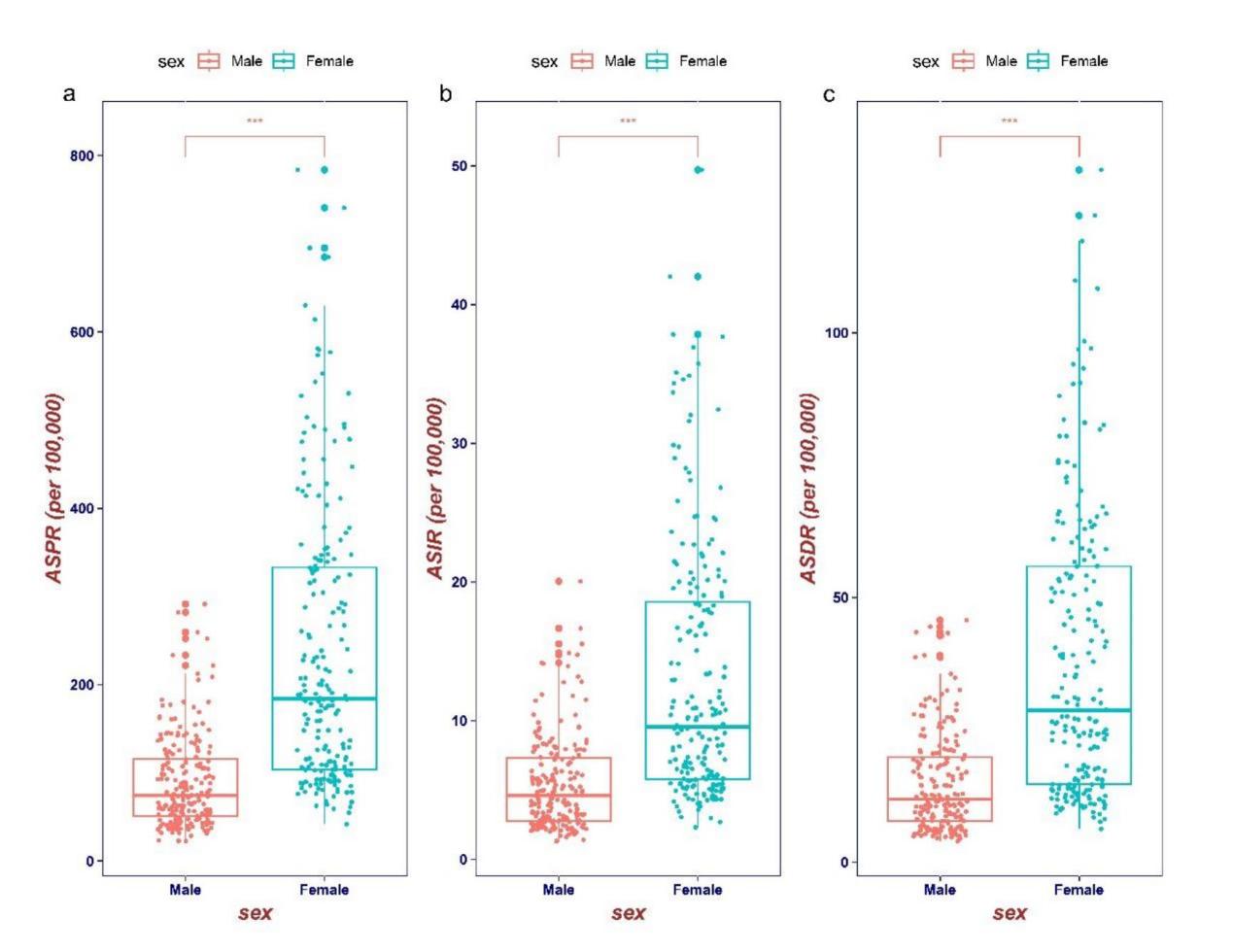


**Figure S5**. Differences in ASPR (a), ASIR (b), ASDR (c) for RA by sex in 204 countries and territories in 2021. ASPR, age-standardized prevalence rate; ASIR, age-standardized incidence rate; ASDR, age-standardized DALYs rate; DALYs, Disability-Adjusted Life Years; ***, *P* < 0.001.


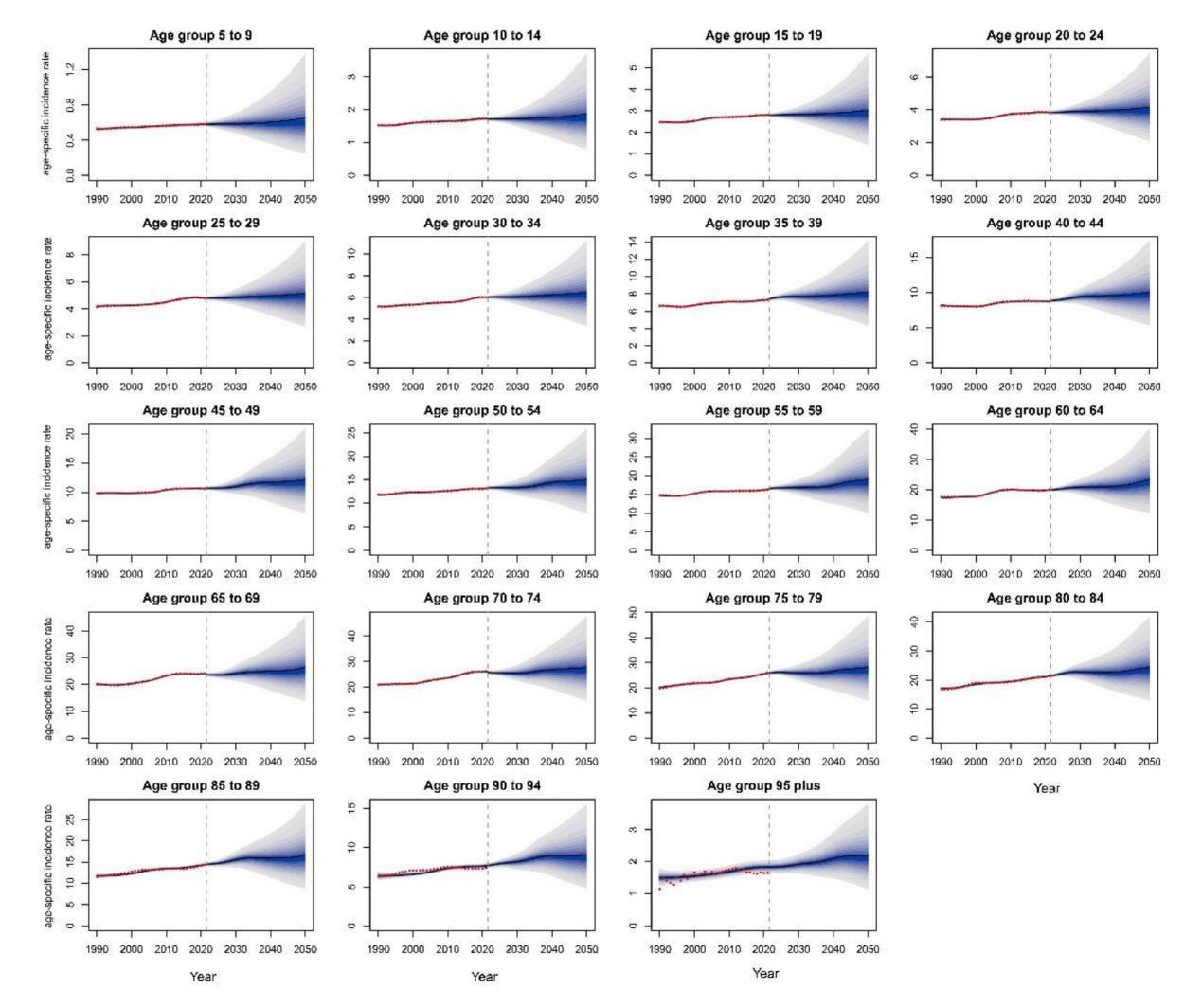


**Figure S6**. Global age-specific incidence rates by age-groups (5 years) of RA from 1990 through 2050 forecasts for males. The shaded area represents 95% CI. RA, rheumatoid arthritis; CI, confidence interval.


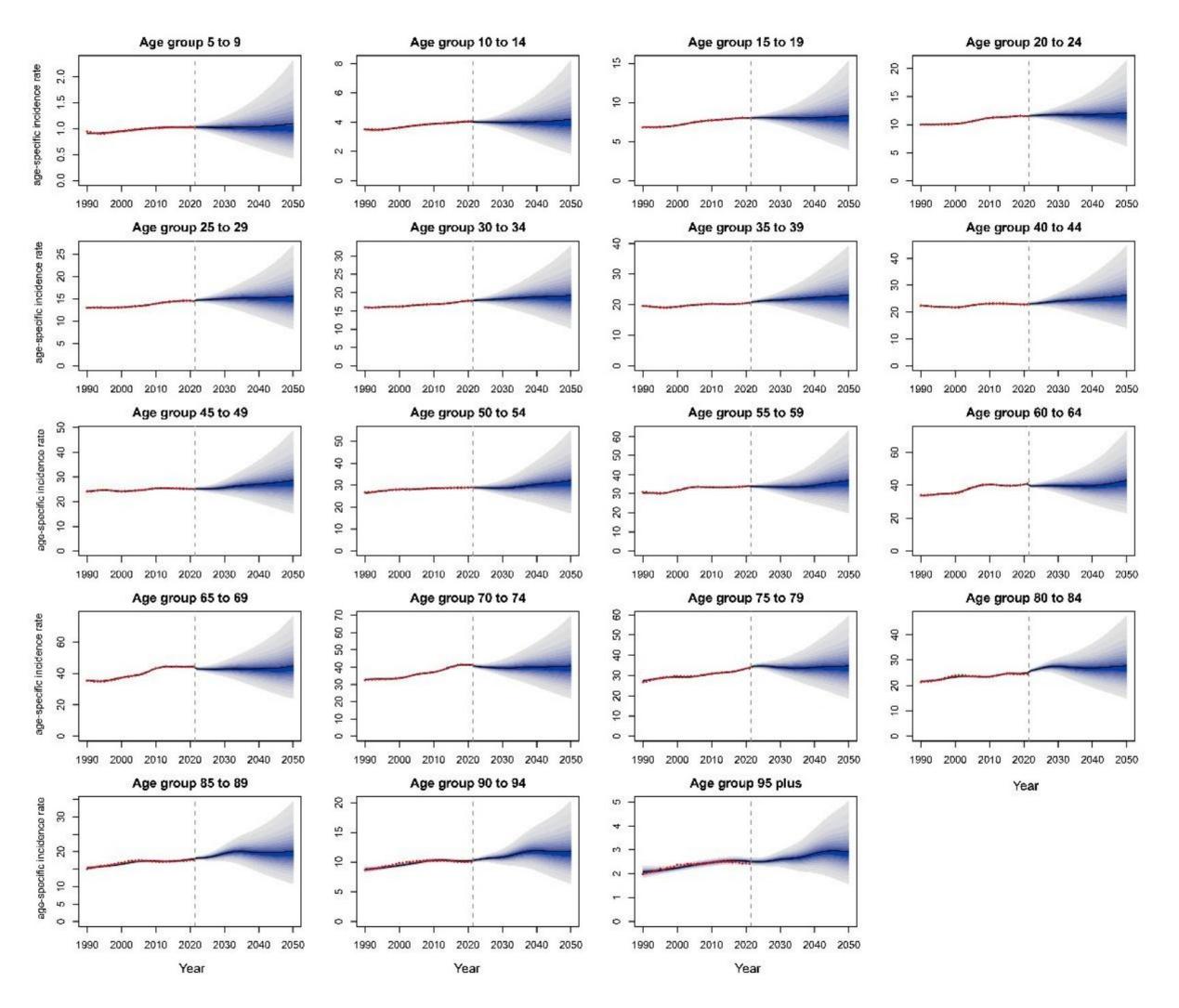


**Figure S7**. Global age-specific incidence rates by age-groups (5 years) of RA from 1990 through 2050 forecasts for females. The shaded area represents 95% CI. RA, rheumatoid arthritis; CI, confidence interval.
